# Electrochemical Immunosensor Based on Polythionine/Gold Nanoparticles for the Determination of Aflatoxin B_1_

**DOI:** 10.3390/s8128262

**Published:** 2008-12-15

**Authors:** Joseph H.O. Owino, Omotayo A. Arotiba, Nicolette Hendricks, Everlyne A. Songa, Nazeem Jahed, Tesfaye T. Waryo, Rachel F. Ngece, Priscilla G.L. Baker, Emmanuel I. Iwuoha

**Affiliations:** SensorLab, Department of Chemistry, University of Western Cape, Private Bag X17, Bellville 7535, Cape Town, South Africa

**Keywords:** Immunosensor, Gold nanoparticles, Aflatoxin B_1_, Polythionine, Horseradish peroxidise (HRP)

## Abstract

An aflatoxin B_1_ (AFB_1_) electrochemical immunosensor was developed by the immobilisation of aflatoxin B_1_-bovine serum albumin (AFB_1_-BSA) conjugate on a polythionine (PTH)/gold nanoparticles (AuNP)-modified glassy carbon electrode (GCE). The surface of the AFB_1_-BSA conjugate was covered with horseradish peroxidase (HRP), in order to prevent non-specific binding of the immunosensors with ions in the test solution. The AFB_1_ immunosensor exhibited a quasi-reversible electrochemistry as indicated by a cyclic voltammetric (CV) peak separation (ΔE_p_) value of 62 mV. The experimental procedure for the detection of AFB_1_ involved the setting up of a competition between free AFB_1_ and the immobilised AFB_1_-BSA conjugate for the binding sites of free anti-aflatoxin B_1_ (anti-AFB_1_) antibody. The immunosensor's differential pulse voltammetry (DPV) responses (peak currents) decreased as the concentration of free AFB_1_ increased within a dynamic linear range (DLR) of 0.6 - 2.4 ng/mL AFB_1_ and a limit of detection (LOD) of 0.07 ng/mL AFB_1_. This immunosensing procedure eliminates the need for enzyme-labeled secondary antibodies normally used in conventional ELISA–based immunosensors.

## 1. Introduction

Aflatoxin B_1_ (AFB_1_) is an example of a group of highly toxic difurancoumarin derivatives that are produced by many strains of *Aspergillus flavus* and *A. parasiticus* which often contaminate a variety of food and animal feed stored under temperate and humid conditions favourable to mould growth [1]. The four major aflatoxins have been designated as B_1_, B_2_, G_1_ and G_2_ based on their fluorescence under UV light and their relative chromatographic mobility during thin layer chromatography. AFB_1_ has been classified as a group 1 human carcinogen and aflatoxins G_1_, G_2_ and B_2_ belong to a group human carcinogens [2]. These toxins exhibit carcinogenic, teratogenic and mutagenic properties and have now been isolated from a wide variety of agricultural products [3]. AFB_1_ can enter the food chain mainly through the ingestion of contaminated human or animal food. The intake of AFB_1_ over a long period of time, even in low concentrations, may be very deleterious to health [4]. The Food and Agricultural Organization 2004 report on mycotoxins [5] revealed that as of December 2003, at least 99 countries worldwide had regulations in place for permitted mycotoxin levels in food/or feed, and have set limits for AFB_1_ alone or for the sum of aflatoxins B_1_, B_2_, G_1_ and G_2_. The maximum permissible level for AFB_1_ in food was set at 2 µg/kg (2 ppb).

Many analytical methods have been developed for the determination of aflatoxins. These include thin-layer chromatography (TLC) [6] and high-performance liquid chromatography (HPLC) [7]. Though these techniques have excellent sensitivities they typically require skilled operators, extensive sample pre-treatment and expensive equipment [8]. The goal of more recent studies has been to simplify and expedite the method of detection while attempting to maintain or improve the sensitivity. Among the immunochemical approaches, the enzyme-linked immunosorbent assay (ELISA) method is the most widely applied. Spectrophotomeric ELISAs specific for AFB_1_ [9, 10], total aflatoxins [11, 12] and AFM_1_ [13, 14] have been developed and their simplicity, adaptability and sensitivity have been demonstrated. In order to achieve higher sensitivity and move to the use of disposable probes, electrochemical immunosensors for aflatoxins based on indirect competitive ELISA format have been proposed [15-17]. These immunosensors require the use of labeled secondary antibodies for detection. To achieve label-free immunosensors, direct electrochemical immunosensors for AFB_1_ based on electrochemical impedance spectroscopy [18], optical waveguide lightmode spectroscopy [19] and room temperature ionic liquids [20] have been reported.

The search for a simple and label-free amperometric immunosensor is of considerable interest. Among the various conducting polymers, thionine (phenothiazine) is a redox dye which has been studied extensively due to its potential utility in sensor applications [21, 22]. Its electroactivity lies not only in the heterocyclic nitrogen atoms and nitrogen bridges, but also in its free amine groups [23]. In addition polythionine (PTH) can be easily functionalised due to the abundant amino groups which adsorb metal ions and various organic halogen substances, thus preventing proteins from damage [24].On the other hand, gold nanoparticles (AuNPs) have been extensively used as matrices for the immobilization of macromolecules such as proteins, enzymes and antibodies; as well as chemical labels for biomolecules [25-27]. Modification of electrode surfaces with AuNPs provides a microenvironment similar to what obtains under physiological conditions [28]. In this investigation, an electrochemical immunosensor for the detection of AFB_1_ was developed by the drop-coating of AuNPs on PTH-modified glassy carbon electrode (GCE) surface. Subsequently AFB_1_-conjugate was adsorbed on to the gold nanoparticles surface. Details of the preparation, characterization and application of the immunosensor are described.

## 2. Experimental

### 2.1 Reagents and materials

Analytical reagent grade chemicals from Sigma-Aldrich were used in all experiments. Phosphate buffer saline (PBS) solution pH 7.2 contained 0.1 M KH_2_PO_4_, 0.1 M Na_2_HPO_4_, 2.7 mM KCl and 0.137 M NaCl. Acetate buffer (pH 6.5) was prepared from 0.1 M CH_3_COONa, 0.1 M CH_3_COOH and 0.1 M KCl. 1 mg/mL Aflatoxin B_1_ (AFB_1_) solution was prepared by dissolving AFB_1_ from *Aspergillus flavus* in methanol followed by dilution in PBS/10% methanol to give a series of standard solutions with a concentration range of 0.1- 3.0 ng/mL. The antibody reagent was an immunoglobin (Ig) fraction of rabbit antiserum AFB_1_ (anti-AFB_1_) antibody that contained 6.8 mg/mL of total protein (which has reactivity with aflatoxins B_1_, G_1_ and B_2_, but no cross reactivity with B_2a_, G_2_, G_2a_ or M_1_) and 0.15 M NaN_3_ as preservative. The anti-AFB_1_ antibody solution for immunosensing reactions was prepared by diluting the stock 6.8 mg/mL solution to 1:2000 v/v in PBS and stored at -20 ^°^C when not in use. Aflatoxin B_1_-BSA conjugate (8–12 mol AFB_1_ per mol BSA), horseradish peroxidase (HRP; 169 units/mL lyophilised powder; EC.1.11.1.7; M_r_ 4000), thionine acetate (3,7-diamino-5-phenothiazinium acetate; C_12_H_9_N_3_S.C_2_H_4_O_2_; M_r_ 287.34), tetrachloro-auric acid (HAuCl_4_.3H_2_0; M_r_ 393.83), sodium citrate and 30% hydrogen peroxide solution were other reagents used in the studies. Colloidal gold nanoparticles (AuNP) (diameter = 20 nm) were prepared according to the procedure of Yuan et a., 2004 [29] by adding 2 mL of 1% (w/w) sodium citrate to a boiling solution of 50 mL 0.01% (w/w) tetrachloro-auric acid. The AuNP solution was stored in a refrigerator in a dark-colored glass bottle. The production of colloidal AuNP was confirmed by UV-Vis measurement covering 200 – 700 nm at room temperature using distilled water as the reference, which gave a maximum absorption at 520 nm. The particle size of the AuNP was determined with transmission electron microscopy (TEM), in which a sample of the colloidal AuNP was dropped on a carbon-coated copper grid and left to dry for 24 h, after which TEM images were recorded and analysed.

### 2.2 Instrumentation

All electrochemical experiments were performed with a BAS/50W integrated automated electrochemical workstation (Bioanalytical Systems Lafayette, IN, USA). Cyclic voltammetry and differential pulse voltammetry experiments were performed with Ag/AgCl (3 M NaCl type) and platinum wire as reference and auxiliary electrodes, respectively. A BAS 3-mm diameter GCE in bare or modified form, was used as the working electrode. Transmission electron microscopy studies were carried out with JEOL JEM-1200 EX II electron microscope. UV-Vis experiments were performed with GBC UV/Vis 920 spectrophotometer (GBC Scientific Instruments, Australia).

### 2.3 Immunosensor preparation

**Scheme 1.**
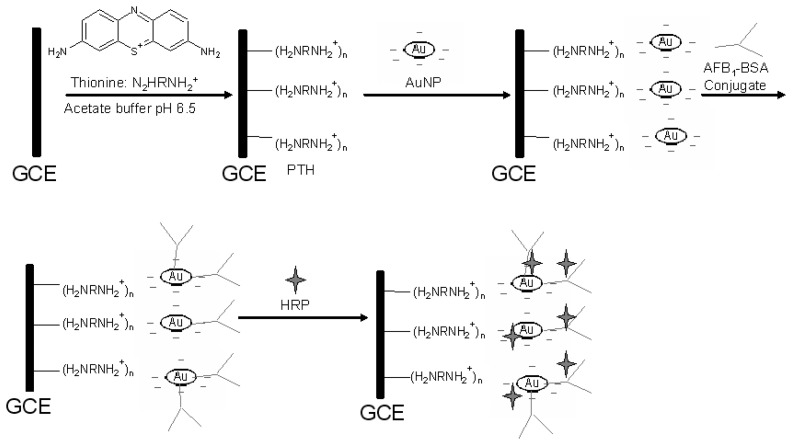
Reaction scheme for the preparation of aflatoxin B_1_ immunosensor. Step 1: polymerization of thionine. Step 2: formation of AuNP layer. Step 3: loading of AFB_1_-BSA conjugate. Step 4: blocking of AFB_1_-BSA conjugate layer with HRP.

Before use the GCE was polished consecutively with 1.0, 0.3 and 0.05 micron aqueous slurry of alumina micropolish (Buehler, IL, USA), followed by sonication in double-distilled water and ethanol for 5 min and dried in air. 0.1 mM thionine was polymerised on the GCE by cyclic voltammetry (CV) in a 20-voltammetric cycle experiment covering a potential window of -400 to +1200 mV at a scan rate of 50 mV/s and a sensitivity of 0.001 A/V to produce the required PTH. Freshly prepared PTH film was drop-coated with 2 µL of colloidal AuNP and allowed to dry for 24 h. Subsequently the electrode was coated with 5 µL of AFB_1_-BSA conjugate (1 µg/mL) and incubated for 1 h at 37 ^°^C. The resultant immunosensor was incubated in 1 mg/mL HRP contained in acetate buffer (pH 6.5) for 60 min at 4 ^°^C in order to block any remaining active sites of the AuNP layer and avoid non-specific adsorptions. The immunosensor was stored at 4 ^°^C when not in use. The schematic illustration of the stepwise immunosensor assembly procedure is shown in Scheme 1.

### 2.4 Procedure for electrochemical immunosensing

Competitive immunosensing was performed in the absence and presence of free AFB_1_. Experiment in the absence of free AFB_1_ (i.e. 0.0 ng/mL AFB_1_) were performed by placing 5 µL of anti-AFB_1_ antibody on top of the immunosensor (i.e. GCE|AuNP|PTH|AFB_1_-BSA-conjugate HRP) and allowed to react for 15 min. The electrode system was immersed in a 1 mL cell solution containing acetate buffer pH 6.5 and 3.2 µM H_2_O_2_ and DPV measurement was performed by scanning cathodically from 0 to -480 mV at 10 mV pulse amplitude, 50 ms pulse width and 20 mV/s potential scan rate. For experiments in the presence of free AFB_1_ (i.e. 0.6 – 3.0 ng/mL AFB_1_), 10 µL of anti-AFB_1_ antibody solution was mixed with 10 µL of 0.6, 1.2, 1.8, 2.4 or 3.0 ng/mL AFB_1_ solutions. 5 µL of this mixture was placed on top of the immunosensor and allowed to react for 15 min. This was followed by DPV measurement as described above for 0.0 ng/mL AFB_1_.

## 3. Results and Discussion

### 3.1 Characterisation of gold nanoparticles

**Figure 1.**
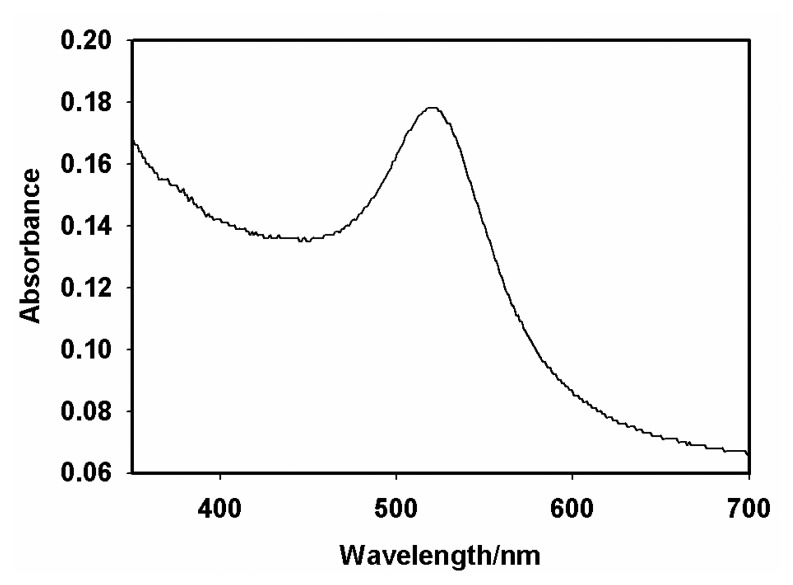
UV-Vis spectrum of colloidal gold nanoparticles.

**Figure 2.**
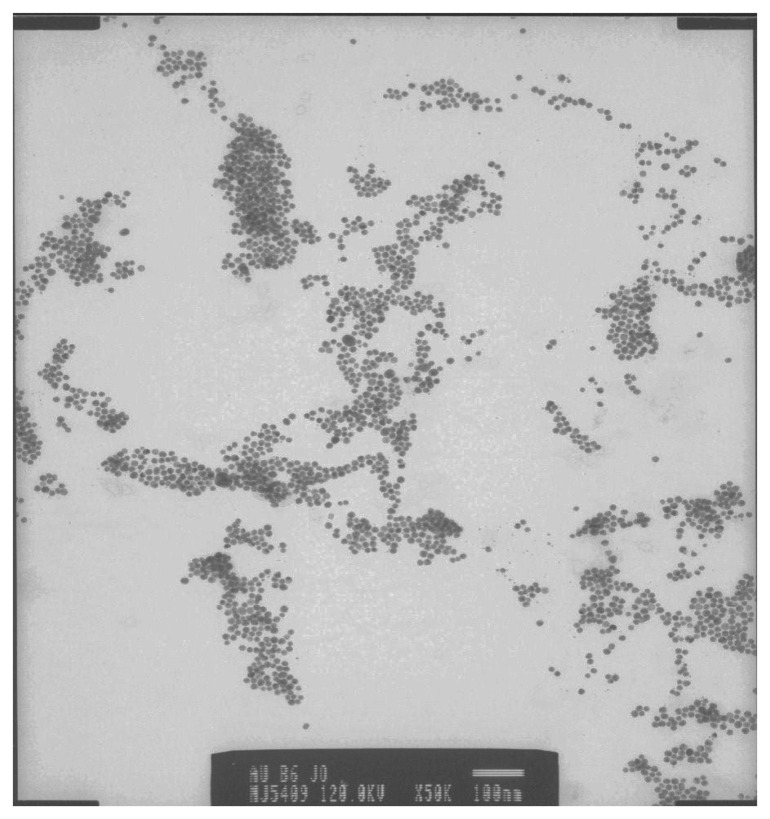
TEM image of colloidal gold nanoparticles.

UV-Vis spectrum of the colloidal gold nano-particles prepared by the method of Yuan *et al.* [29] showed a maximum absorption, λ_max_, at 520 nm (Figure 1). The formation of nano-particles and the particle size (20 nm diameter) were confirmed by the TEM data (Figure 2). These results agreed with what has been reported for colloidal AuNP [29].

### 3.2 Electropolymerization of multiporous thionine film

**Figure 3.**
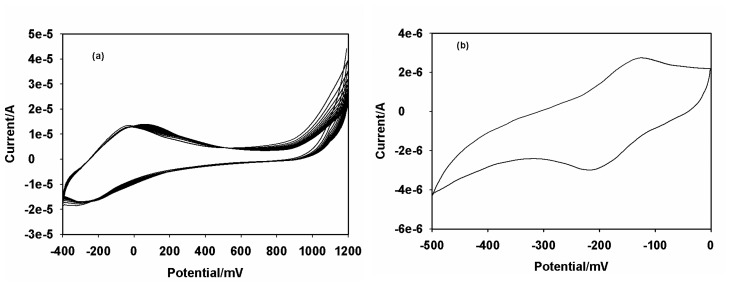
Cyclic voltammograms (a) for the synthesis of PTH from 0.1 mM thionine in acetate buffer (pH 6.5); and (b) of GCE|PTH in acetate buffer (pH 6.5). (Scan rate: 50 mV/s)

**Figure 4.**
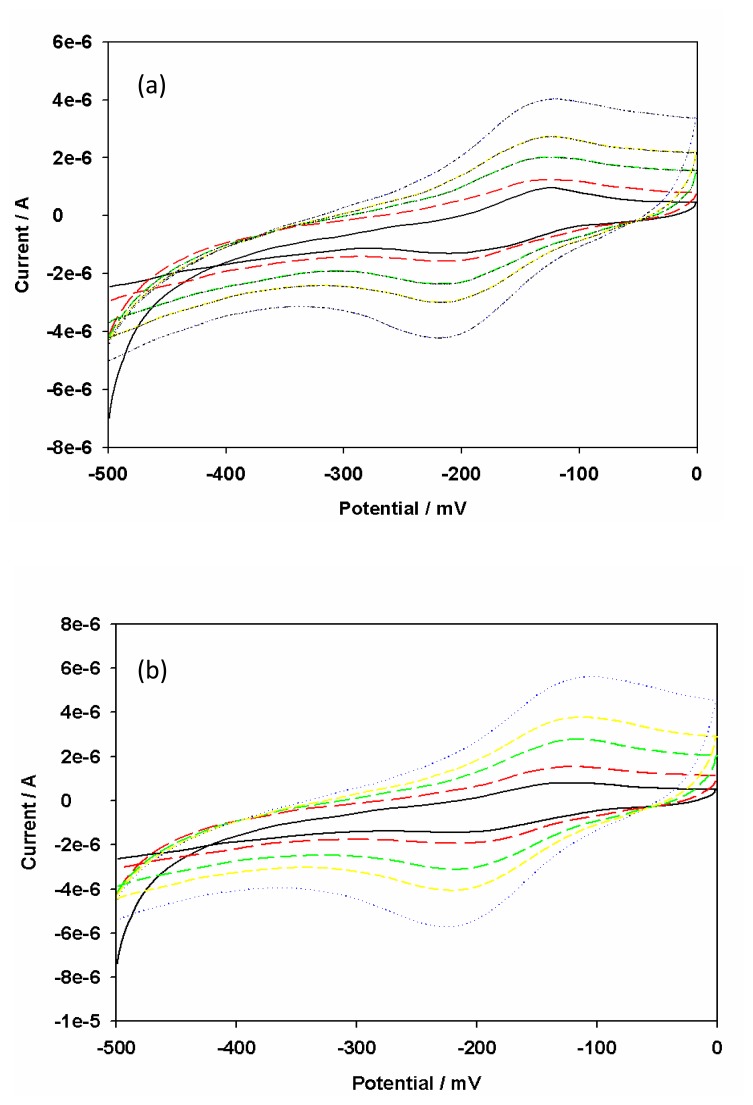
CV's of **(a)** PTH|GCE and **(b)** GCE|PTH|AuNP|AFB_1_-BSA-conjugate in acetate buffer (pH 6.5) at 5, 10, 20, 30 and 50 mV/s (starting from the inner CV).

The GCE was first reduced in acetate buffer solution (pH 6.5) containing thionine at -1500 mV to make it negatively charged and interact with positively charged thionine in the mildly acidic conditions. Subsequently cyclic voltammetry was performed at a bias voltage of -400 to +1200 mV at a scan rate of 50 mV/s. When the applied potential exceeded +1100 mV, the electropolymerization reaction proceeded and formed cationic radical species on the GCE [30]. This process resulted in a multiporous structure which facilitated the assembly of AuNP. The oxidation potential in the first step was the most important factor for electropolymerization of thionine and should not be less than +1100 mV. Two reasons suffice for this. Firstly in order to achieve the formation of polythionine (PTH) film, the electrode potential must be larger than the potential at which the oxidations of –NH_2_ groups of thionine molecule occurs, and secondly the modified cation must be necessarily associated with the surface activation of GCE. During thionine electropolymerization, a pair of quasi reversible redox peaks with a cathodic peak potential (E_pc_) of -250 mV and an anodic peak potential E_pa_ of -100 mV, increased gradually and tended to become stable with increasing number of polymerization cycles (Figure 3a). On removal of the electrode from the dye-containing solution, a golden film was seen on the electrode surface. The CV of the GCE|PTH in acetate buffer (pH 6.5) (Figure 3b) confirmed the presence of surface-bound electroactive material. Figure 4 shows the scan rate dependence of the electrochemistry of GCE|PTH and GCE|PTH|AuNP|AFB_1_-BSA-conjugate electrodes in acetate buffer pH 6.5. The peak currents varied linearly with scan rate as is characteristic of the electrochemistry of a surface-bound thin film of electroactive material [31]. It is thus suggested that nearly all the reduced polythionine (PTH_(red)_) was converted to the oxidized polythionine (PTH_(ox)_) on the forward scan and vice versa.

### 3.3 Electrochemical characteristics of the electrode

**Figure 5.**
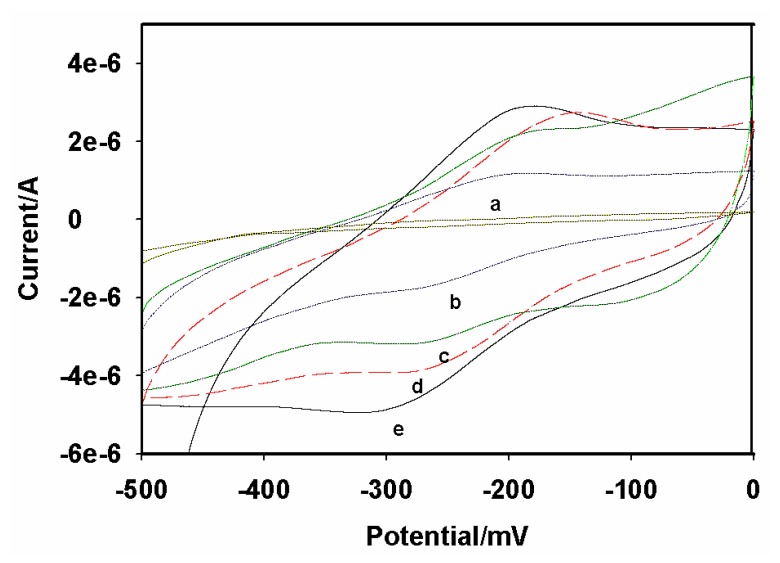
CV's of **(a)** bare GCE; **(b)** GCE|PTH|; **(c)** GCE|AuNP|PTH|AFB_1_-BSA-conjugate|HRP-blocked; **(d)** GCE|AuNP|PTH|AFB_1_-BSA-conjugate and **(e)** GCE|AuNP|PTH. Conditions: acetate buffer ph 6.5; scan rate = 10 mV/s.

The stepwise assembly of the immunosensor was monitored cyclic-voltammetrically in acetate buffer pH 6.5 and the results are shown in Figure 5. The CV of the bare GCE is shown as Figure 5a. The bare GCE did not exhibit any redox chemistry over the potential window (-500 to 0 mV) used in the study. The GCE|PTH gave a quasi reversible electrochemistry with a peak separation of ∼62 mV at 10 mV/s (Figure 5b). The redox couple can be ascribed to the electrochemistry of the PTH film on the GCE. The peak currents increased after the modification of GCE|PTH with AuNP (Figure 5e). The reason for this increase is that nanometer-sized particles of colloidal gold behave like a conducting wire or electron conducting tunnel, which makes it easier for the electron transfer to take place. However, the peak currents decreased (Figure 5d) after AFB_1_-BSA conjugate was incorporated to produce GCE|PTH|AuNP|AFB_1_-BSA-conjugate, which indicated that the relatively insulating AFB_1_-BSA conjugate was immobilized on the electrode surface. The subsequent blocking of possible active sites with HRP to form GCE|PTH|AuNP|AFB_1_-BSA-conjugate|HRP immunosensor system decreased the CV peak current due to the adsorption of the protein, HRP (Figure 5c). Surface concentration (Γ^*^) values of 7.06 × 10^-11^ and 9.65 × 10^-10^ mol/cm^2^ were calculated for GCE|PTH and GCE|PTH|AuNP|AFB_1_-BSA-conjugate electrode systems, respectively.

### 3.4 Assay of the HRP enzymatic catalytic activity

**Figure 6.**
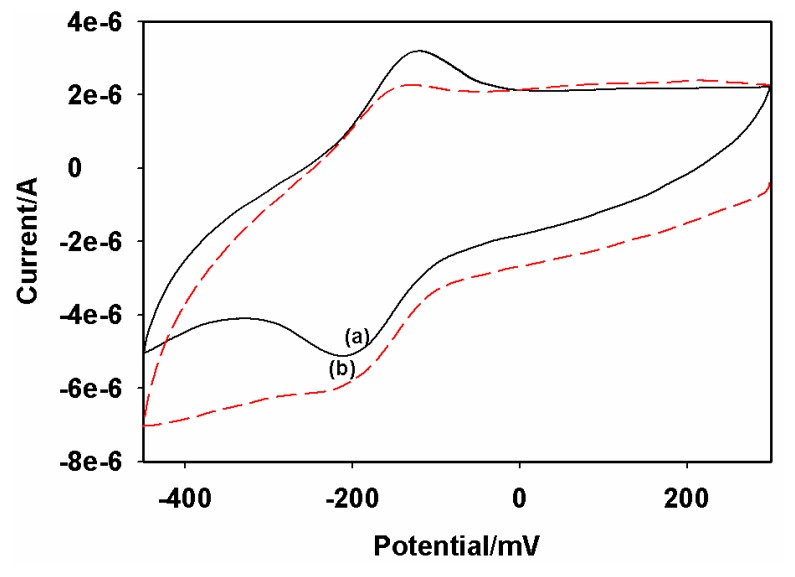
CV's of AFB_1_ immunosensor (GCE|AuNP|PTH|AFB_1_-BSA-conjugate|HRP-blocked) in acetate buffer (pH 6.5): in the absence (a), and presence (b), of 3.2 µM H_2_O_2_. Scan rate = 10 mV/s.

Horseradish peroxidase was used in the preparation of the immunosensors to block possible uncovered active sites on the GCE|AuNP|PTH|AFB_1_-BSA-conjugate in order to avoid non-specific adsorption. Experiments were performed with hydrogen peroxide to ascertain if HRP was indeed immobilized on the electrode. Figure 6 depicts the CV's of the proposed immunosensor in the presence and absence of H_2_O_2_. A redox couple representing the electrochemistry of PTH was observed in the absence of H_2_O_2_ (Figure 6a). However, an enhancement of the cathodic peak and a concomitant decrease of the anodic peak current were observed in the presence of 3.2 µM H_2_O_2_ (Figure 6b). It can thus be concluded that HRP was attached to the immunosensor surface and it retained its enzymatic catalytic activity [21], which is coupled to the PTH electron transfer process shown below.


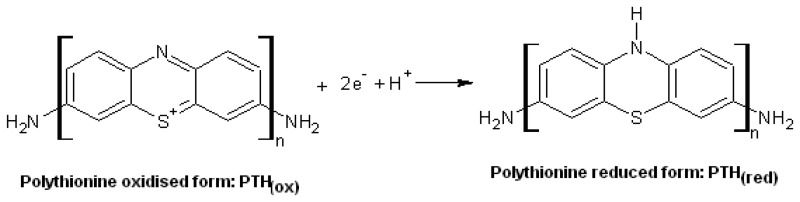


**Figure 7.**
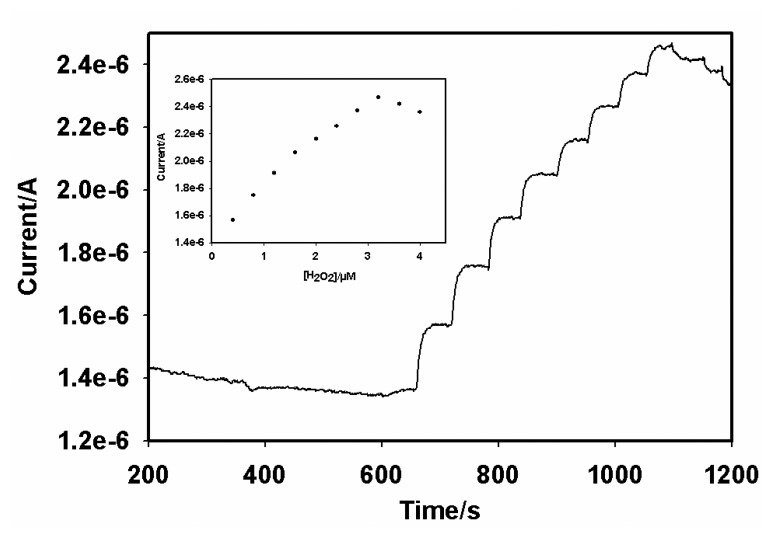
Steady-state amperometric responses of AFB_1_ immunosensor (GCE|AuNP|PTH|AFB_1_-BSA-conjugate|HRP-blocked) to H_2_O_2_ in acetate buffer (pH 6.5) at -175 mV. Inset: H_2_O_2_ calibration curve obtained from the steady state responses.

The optimal amount of H_2_O_2_ required for the immunosensing reaction with GCE|AuNP|PTH|AFB_1_-BSA-conjugate|HRP-blocked electrode system was determined from steady-state amperometry experiments performed at -175 mV. As shown in Figure 7, the sensor gave a maximum response at 3.2 µM H_2_O_2_. At H_2_O_2_ concentrations higher than 3.2 µM, the sensor response decreased owing to the irreversible transition of the immobilized HRP to its inactive form [32]. As a result, 3.2 µM H_2_O_2_ was chosen for the immunosensing experiments.

The effect of pH on the immunosensor was studied for pH's 4.5 to 6.5 at 25 ^°^C. In order to retain the bioactivity of both AFB_1_-BSA conjugate and HRP, pH 6.5 was chosen as the optimal pH. Although 37 ^°^C is the best incubation temperature for antibody-antigen reactions, immunoproteins and HRP do not maintain their activities for a long time at this temperature. As a result 25 ^°^C was used in all experiments.

### 3.4 Performance of the immunosensor

**Figure 8.**
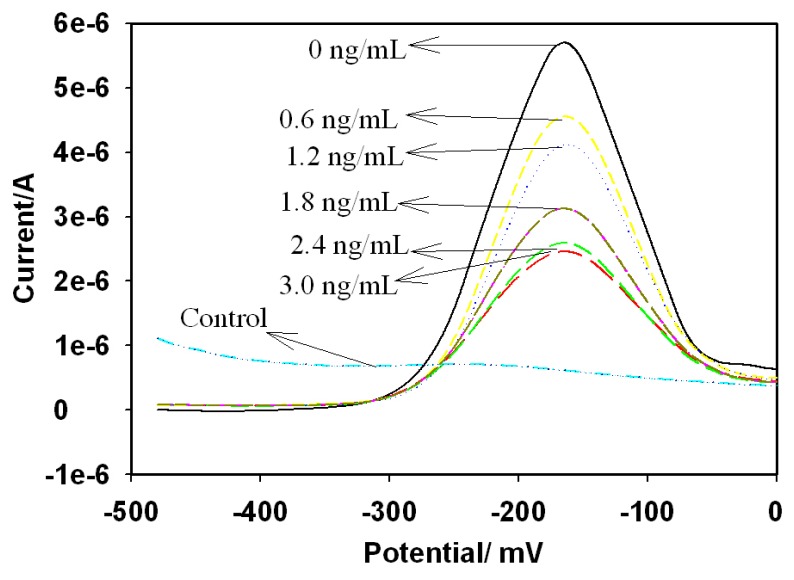
DPV responses of AFB_1_ immunosensor (GCE|AuNP|PTH|AFB_1_-BSA-conjugate|HRP-blocked), for 0 - 3 ng/mL AFB_1_ (mixed with anti-AFB_1_ antibody) in acetate buffer (pH 6.5) containing 3.2 µM of H_2_O_2_. DPV experimental conditions: scan rate = 20 mV/s; pulse amplitude = 10 mV; and pulse width = 50 ms.

The responses of the immunosensor to AFB_1_ were recorded with DPV as shown in Figure 8 for 0 – 3 ng/mL AFB_1_. As expected, the peak current was inversely proportional to the analyte concentration. The detection principle is based on the inhibition of the active centre of HRP by the formation of antigen-antibody complex. The observed current is attributed to the catalytic response of the adsorbed HRP to H_2_O_2_. The formation of the antigen-antibody complex introduces a local current change at the sites of the adsorbed HRP due to the inhibition of the H_2_O_2_ reduction reaction. The attenuation of the DPV response is dependent on AFB_1_ concentration as shown in Figure 8 and it is attributable to the increase in electron transfer resistance of adsorbed HRP [33] caused by the insulating properties of the complex formed by the binding of AFB_1_-BSA conjugate and anti-AFB_1_ antibody.

**Figure 9.**
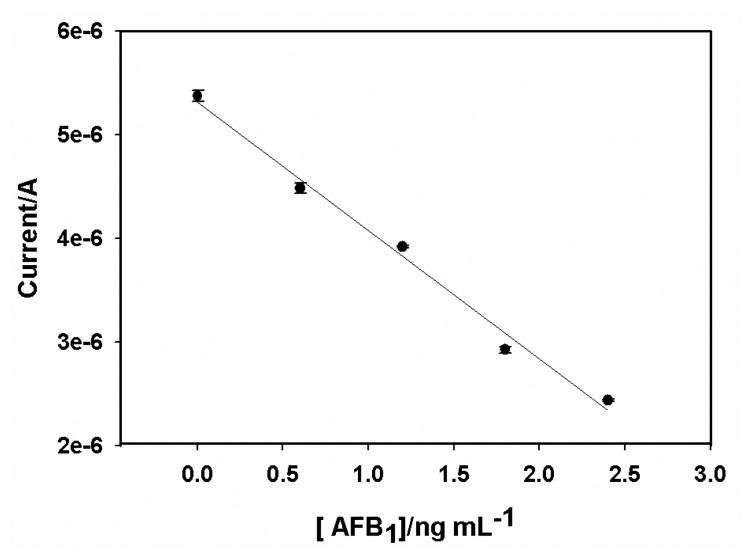
Calibration plot of AFB_1_ immunosensor. Conditions are as given in Figure 8.

The calibration graph of the AFB_1_ immunosensor (Figure 9) plotted from DPV results gave DLR, sensitivity and LOD values of 0.6 - 2.4 ng/mL, 1.23 x 10^-6^ A/(ng/mL) and 0.07 ng/mL, respectively. The DPV measurements were carried out in triplicates. The relative standard deviations of the DPV responses were 3.1%, 2.9%, 2.4% and 5.1% for 0.6, 1.2, 1.8 and 2.4 ng/mL, respectively, indicating acceptable level of precision.

## 4. Conclusions

The DLR and LOD of the immunosensor were compared with those of other electrochemical AFB_1_ immunosensors [15, 18, 20] as shown in Table 1. The values of these sensor parameters for the PTH-based immunosensor are in good agreement with those reported by other laboratories. In addition, the sensor exhibited high sensitivity and good reproducibility. These characteristics of the immunosensor show that it can be used to screen food products for AFB_1_, since the DLR and LOD values cover the FAO's limit of 2 ppb AFB_1_ in food samples [5]. The immunosensing procedure reported in this study eliminated the requirement of secondary labeled antibodies as is the case in conventional electrochemical immunosensors based on ELISA techniques.

## Figures and Tables

**Table 1.** Comparison of the analytical parameters of AFB_1_ immunosensor.

**Immunosensor**	**DLR (ng/mL)**	**LOD (ng/mL)**	**Reference**
96-well screen printed microplate	0.05 – 2	0.03	[15]
Pt|PSSA|PANi|Anti-AFB_1_	0.1 - 0.6	0.1	[18]
GCE|Nafion|RTIL|TiO_2_|AuNP|Anti-AFB_1_-HRP	0.1 – 12	0.05	[20]
GCE|AuNP|PTH|AFB_1_-BSA-conjugate|HRP-blocked	0.6 - 2.4	0.07	This work
